# Editorial comment: Findings regarding non-sexual penile fracture in a referral emergency hospital

**DOI:** 10.1590/S1677-5538.IBJU.2020.0420.2

**Published:** 2021-02-03

**Authors:** Eduardo B. Bertero

**Affiliations:** 1 Hospital do Servidor Público Estadual Seção de Medicina Sexual Departamento de Urologia São Paulo SP Brasil Departamento de Urologia, Seção de Medicina Sexual, Hospital do Servidor Público Estadual, São Paulo, SP, Brasil.

## COMMENT

This paper called my attention as the number of patients studied on this very uncommon pathology is incredibly significant. The authors gathered, retrospectively, 149 men with a history of penile fracture (PF) who sought the emergency room of a very high-volume public hospital in Brazil, between January 2014 and January 2019 (1). I did not find any paper with such a considerable population published, at least recently on the literature. All cases were operated on immediately by a Urologist and his Resident on duty. The penis was degloved after a circumcision and the hematoma was evacuated and the tear on the tunica albuginea identified and repaired. If a urethral lesion was found, it was treated with a simple interrupted closure under a Foley catheter.

Of a total of 149 patients submitted to surgical treatment for PF, 18 (12%) reported non-sexual cause. Twelve (66.6%) cases were due to penile manipulation through the act of bending the penis during morning erection, three (16.6%) when rolling over in bed with erect penis, one (5.5%) when embracing the wife during erection, one (5.5%) to laying on the partner with erect penis and the other (5.5%) when sitting on the toilet with an erection.

A recent metanalyses identified a new subpopulation associated with non-sexual activity penile fracture: injection of collagenase Clostridium histolyticum used in the treatment of Peyronie's disease (2). We do not have this treatment option available in Brazil.

The authors report using ultrasound imaging only on a few cases who the diagnosis was difficult. They did not have available, magnetic resonance imaging, although it should be the first tool used on this condition (3). I understand that the authors were indeed very lucky relying on just this tool, and did not find any “false” penile fracture, such as dorsal vein rupture, although this should be considered a surgical emergency as well (4).

It would be interesting to know the relationship between the hours of evolution of the penile fracture until the surgery was performed, and the progression with erectile dysfunction and formation of nodules and plaques. The literature reports it is particularly important to operate as soon as possible any case of penile fracture. Delay more than 24 hours was found to be associated with wound infection and ED (5, 6). The authors report that they operated on every case, regardless of the timing of the occurrence. Sharma and cols reported on a study of 68 men who fractured their penis during sexual activity, and found that age (>50 years) and bilateral corporal involvement were risk factors for development of ED post-operatively (7).

Finally, the authors found that in the Brazilian population there is not the attitude of bending their own penis to achieve detumescence. In their series, only one case of sitting on the toilet with erect penis. This is a quite common habit in the middle east countries called Taghaandan (8).

The present study has some limitations as part of the data was obtained retrospectively. However, to my knowledge this is the first study to address the findings and outcomes of PF specifically and exclusively with non-sexual etiology in a latin american country. Congratulation to the authors.
